# Application of Japanese guidelines for gestational weight gain to multiple pregnancy outcomes and its optimal range in 101,336 Japanese women

**DOI:** 10.1038/s41598-019-53809-8

**Published:** 2019-11-21

**Authors:** Kyoko Nomura, Kengo Nagashima, Shunji Suzuki, Hiroaki Itoh

**Affiliations:** 1 Department of Environmental Health Science and Public Health, Akita University Graduate School of Medicine, Akita City, 010-8543 Japan; 2 Research Center for Medical and Health Data Science, The Institute of Statistical Mathematics, Tachikawa City, 190-0862 Japan; 3 Department of Obstetrics and Gynecology, Japanese Red Cross Katsushika Maternity Hospital, Katsushika Ku, 124-0012 Japan; 4 Department of Obstetrics and Gynecology, Hamamatsu University School of Medicine, Hamamatsu, 431-3192 Japan

**Keywords:** Epidemiology, Reproductive signs and symptoms

## Abstract

This study was performed to investigate whether the Japanese guidelines for gestational weight gain (GWG) can be used to determine the risks of multiple pregnancy outcomes and estimate optimal GWG in 101,336 women with singleton pregnancies in 2013. Multivariable logistic regression analyses indicated that the risks associated with low birth weight, small for gestational age, and preterm birth increased significantly with weight gain below the Japanese guidelines, and the risks of macrosomia and large for gestational age increased with weight gain above the guidelines regardless of Asian-specific pre-pregnancy body mass index (BMI). The GWG cutoff points estimated from the adjusted area under the receiver operating characteristics curve >0.6 corresponded to 10–13.8 kg in underweight women with pre-pregnancy BMI < 18.5 kg/m^2^; 10–13.7 kg in normal weight women with pre-pregnancy BMI 18.5–22.9 kg/m^2^; 8.5–11.4 kg in overweight women with pre-pregnancy BMI 23–24.9 kg/m^2^, 5–13.3 kg in obese women with pre-pregnancy BMI 25–29.9 kg/m^2^; and 4.7 kg in obese women with pre-pregnancy BMI ≥ 30 kg/m^2^. The optimal GWG ranges proposed by the present study are slightly higher than those recommended by the current Japanese guidelines.

## Introduction

In Japan, one quarter of women of reproductive age are underweight, as defined by body mass index (BMI) < 18.5 kg/m^2^, and underweight women are at risk of delivering low-birth-weight (LBW) infants^1,2^. Recent studies have implied that epigenetic alterations are induced by the uterine environment of underweight mothers. The “developmental origins of health and disease” hypothesis^3^ proposes that fetal undernutrition has permanent effects on growth and can cause lifestyle-related diseases such as type 2 diabetes and cardiovascular disease^4^.

In 2018, the incidence of LBW infants was higher in Japan (9.4%) than the average among Organization for Economic Cooperation and Development (OECD) countries (6.5%)^5^. This high incidence of small babies has been observed for over two decades in Japan^6^, and pre-pregnancy underweight mothers and poor weight gain during pregnancy are major related factors^7^. The Japanese government has taken measures to reduce the number of LBW infants since 2001 in a national campaign to promote maternal and child health in the 21^st^ century, but the incidence increased between 2001 and 2014 and is still far above the average of OECD countries^8^.

The Japanese Ministry for Health, Labor, and Welfare introduced gestational weight gain (GWG) recommendations in 2001 for appropriate weight gain during pregnancy^9^: 9–12 kg for underweight and 7–12 kg for normal-weight women, and ≤5 kg (depending on individual cases) for overweight and obese women. A recent study^10^ that investigated attitudes to strict adherence to the GWG guidelines reported that 80% of pregnant women considered avoiding excessive GWG important “for ease of delivery and/or her health.” However, those who limited GWG to below or within the guidelines were more likely to have LBW infants than were those whose GWG exceeded the guidelines. A study^2^ of 97,157 pregnant Japanese women using the Japanese Society of Obstetrics and Gynecology (JSOG) Successive Pregnancy Birth Registry System showed that the average weight gain during pregnancy was 10.3 kg in underweight and 10.1 kg in normal-weight mothers, implying that both obstetricians and pregnant women in Japan follow the government guidelines.

Several studies have investigated the combination of pre-pregnancy BMI and total GWG associated with pregnancy outcomes with various assessments, including statistical interactions^11^, stratification by pre-pregnancy BMI level^2,12,13^, and the speed of weight gain^14–17^. Furthermore, some recent studies investigated the applicability of the Institute of Medicine (IOM)^18^ guidelines to Asian women^2,17^. However, the guidelines^18^ were developed for safe pregnancy outcomes in Western countries, where the prevalence of obesity is higher. Excessive weight gain in small and lean Asian women may result in additional adverse events, such as excessive hemorrhage and pregnancy-induced hypertension^19^, and therefore the applicability of the IOM guidelines to Asian women still requires scientific justification^20^.

We previously investigated whether the IOM and Japanese guidelines could identify the risks of small for gestational age (SGA) and large for gestational age (LGA) births associated with GWG below and above the recommended level and estimated the optimal GWG in a population of 8,152 Japanese women recruited at a single hospital^21^. The present study was performed to investigate whether the current Japanese GWG recommendations can significantly determine the risks of multiple pregnancy outcomes using the World Health Organization (WHO) Asian-specific pre-pregnancy BMI classification^22^, and to estimate the optimal weight gain range using a GWG cutoff point associated with the risk of multiple pregnancy outcomes by multivariate adjusted receiver operating characteristic (ROC) curve analysis^23^. We focused specifically on the Japanese guidelines, as the incidence of LBW infants in Japan has been high among OECD countries for over two decades, and scientific evidence is urgently required to determine whether the current guidelines should be changed.

## Results

Table 1 shows the basic characteristics of the study population according to the WHO Asian-specific pre-pregnancy BMI classification. Among 101,336 subjects included in the analyses, 18.1% were underweight (pre-pregnancy BMI < 18.5 kg/m^2^, mean age 31.0 years), 9.9% were overweight (pre-pregnancy BMI 23–24.9 kg/m^2^, mean age 31.9 years), 7.8% were obese I (pre-pregnancy BMI 25–29.9 kg/m^2^, mean age 32.6 years), and 3.1% were obese II (pre-pregnancy BMI ≥ 30 kg/m^2^, mean age 32.3 years). The mean (SD) GWG was 10.2 (3.7) kg among underweight women, 10.2 (3.9) kg among normal-weight women, 9.5 (4.5) kg among overweight women, 7.9 (5) kg among obese I women, and 5.2 (5.7) kg among obese II women. These values for underweight and normal-weight women fell into the range recommended by the Japanese Ministry of Health, Labour, and Welfare, but those for overweight and obese women exceeded the recommended ranges. Underweight women tended to have SGA and LBW infants, whereas overweight and obese women tended to have LGA infants, macrosomia, gestational diabetes mellitus (GDM), pregnancy-induced hypertension, and postpartum hemorrhage with cesarean section (PPH-CS).

**Table 1:** Basic characteristics according to WHO Asian-specific BMI classification.

	Asian specific pre-pregnancy BMI									
	<18.5 kg/m^2^		18.5–22.9 kg/m^2^		23.0–24.9 kg/m^2^		25–29.9 kg/m^2^		30 - kg/m^2^	
	Underweight		Normal weight		Overweight		Obese I		Obese II	
	n = 18,382		n = 61,960		n = 9,985		n = 7,873		n = 3,136	
	n or mean	(%) or SD	n or mean	(%) or SD	n or mean	(%) or SD	n or mean	(%) or SD	n or mean	(%) or SD
Age, years	31.0	5.4	31.9	5.3	32.4	5.5	32.6	5.5	32.3	5.3
Maternal height, cm	158.6	5.4	158.2	5.4	157.7	5.5	157.8	5.7	158.1	5.8
Pre-pregnancy weight, kg	44.3	3.6	51.2	4.5	59.4	4.3	67.3	5.9	84.3	10.1
Pre-pregnancy BMI, kg/m^2^	17.6	0.8	20.4	1.2	23.9	0.6	27	1.4	33.7	3.4
Weight at delivery	54.5	5.2	61.4	6.1	68.9	6.5	75.2	7.7	89.7	11.1
BMI at delivery	21.7	1.7	24.5	1.9	27.7	1.9	30.2	2.3	35.9	3.7
Gestational week at delivery	38.2	2.2	38.4	2.2	38.3	2.2	38.2	2.4	38.1	2.6
Gestational weight gain	10.2	3.7	10.2	3.9	9.5	4.5	7.9	5	5.2	5.7
Nulliparity	9,938	(54.2)	31,669	(51.2)	4,653	(46.8)	3434	(43.7)	1475	(47.1)
**Delivery Method**										
Vaginal Delivery	14,089	(76.7)	45,426	(73.3)	6,685	(67.0)	4,913	(62.4)	1,758	(56.1)
Caesarean Section	4,155	(22.6)	16,027	(25.9)	3,221	(32.3)	2,889	(36.7)	1,341	(42.8)
Other method	138	(0.8)	507	(0.8)	79	(0.8)	71	(0.9)	37	(1.2)
**Smoking habits**										
Never	16,800	(91.4)	57,250	(92.4)	9,070	(90.8)	6,996	(88.9)	2,660	(84.8)
Past	976	(5.3)	3,087	(5.0)	556	(5.6)	505	(6.4)	267	(8.5)
Current	606	(3.3)	1,623	(2.6)	359	(3.6)	372	(4.7)	209	(6.7)
**Pregnancy Outcome**										
SGA	2,290	(12.5)	5,121	(8.3)	706	(7.1)	553	(7.0)	226	(7.2)
Low birth weight	3,635	(19.8)	8,635	(13.9)	1,272	(12.7)	1091	(13.9)	424	(13.5)
Preterm birth	2,205	(12.0)	6,199	(10.0)	1,094	(11.0)	915	(11.6)	393	(12.5)
LGA	885	(4.8)	5,585	(9.0)	1,282	(12.8)	1,295	(16.5)	688	(21.9)
Macrosomia	45	(0.2)	389	(0.6)	102	(1.0)	144	(1.8)	102	(3.3)
GDM	535	(2.9)	2,322	(3.8)	694	(7.0)	1,036	(13.2)	801	(25.5)
Pregnancy -induced Hypertension	613	(3.3)	2,673	(4.3)	674	(6.8)	816	(10.4)	501	(16.0)
PPH with VD	3644	(19.8)	13,156	(21.2)	2,140	(21.4)	1,657	(21.1)	694	(22.1)
PPH with CS	893	(4.9)	4,605	(7.4)	972	(9.7)	910	(11.6)	437	(13.9)

BMI, Body Mass Index; SGA, small for gestational age; LGA, large for gestational age; GDM, Gestational Diabetes Mellitus; PPH with VD, postpartum hemorrhage with vaginal delivery; PPH with CS, postpartum hemorrhage with cesarean section.

Table 2 shows the risks of various pregnancy outcomes associated with GWG below and above the government recommendations comparing with weight gain within the recommendations among underweight and normal-weight women. Compared to women who gained weight within the government recommended range, women with weight gain below the recommended ranges had a significantly increased risk of having small babies (i.e., SGA and LBW) and preterm birth (all adjusted *p* < 0.0001). Women with weight gain above the recommendations had significantly increased risks of LGA, macrosomia, pregnancy-induced hypertension, PPH with vaginal delivery (PPH-VD), and PPH-CS (all adjusted *p* < 0.0001).

**Table 2:** Risks of pregnancy outcomes for underweight and normal-weight women according to GWG below and above the recommendation of the Japanese government comparing GWG within the recommended levels.

Underweight women (n = 18,382): pre-pregnancy BMI < 18.5	Wegith gain during pregnancy recommended by Japanese Ministry of Health, Labour, and Welfare							
	<9 kg (n = 6,417)			9–12 kg (n = 6,995)		12 < kg (n = 4,970)		
	n (%)	Crude OR	Adjusted OR (95% CI)	n (%)	OR	n (%)	Crude OR	Adjusted OR (95% CI)
SGA	1172 (18.3)	1.86	1.87 (1.69–2.07)^a^	752 (10.8)	1	366 (7.4)	0.66	0.63 (0.55–0.72)^a^
Low birth weight	2119 (33.0)	2.65	1.91 (1.72–2.11)^b^	1097 (15.7)	1	419 (8.4)	0.50	0.61 (0.53–0.70)^b^
Preterm birth	1366 (21.3)	2.77	2.85 (2.57–3.17)^c^	623 (8.9)	1	216 (4.4)	0.47	0.43 (0.37–0.51)^c^
LGA	129 (2.0)	0.47	0.45 (0.37–0.56)^d^	290 (4.2)	1	466 (9.4)	2.39	2.48 (2.13–2.89)^d^
Macrosomia	3 (0.1)	0.36	0.48 (0.13–1.78)^e^	9 (0.1)	1	33 (0.7)	5.19	4.64 (2.21–9.78)^e^
GDM	281 (4.4)	1.94	1.89 (1.55–2.30)^f^	161 (2.3)	1	93 (1.9)	0.81	0.84 (0.65–1.09)^f^
Pregnancy-Induced Hypertension	192 (3.0)	1.03	0.76 (0.61–0.94)^g^	203 (2.9)	1	218 (4.4)	1.54	1.71 (1.40–2.09)^g^
PPH with VD	1021 (15.9)	0.73	0.85 (0.78–0.94)^d^	1434 (20.5)	1	1189 (23.9)	1.22	1.11 (1.02–1.22)^d^
PPH with CS	302 (4.7)	0.98	0.66 (0.56–0.78)^e^	335 (4.8)	1	256 (5.2)	1.08	1.32 (1.12–1.57)^e^
**Normal weight women (n = 61,960): pre-pregnancy BMI 18.5–22.9 kg/m**^2^	**Weight gain during pregnancy recommended by Japanese Ministry of Health, Labour, and Welfare**							
	**<7 kg (n = 10,686)**			**7–12 kg (n = 34,172)**		**12 < kg (n = 17,102)**		
	**n (%)**	**Crude OR**	**Adjusted OR (95% CI)**	**n (%)**	**OR**	**n (%)**	**Crude OR**	**Adjusted OR (95% CI)**
SGA	1382 (12.9)	1.62	1.51 (1.41–1.62)^b^	2863 (8.4)	1.00	876 (5.1)	0.59	0.59 (0.55–0.64)^b^
Low birth weight	3131 (29.3)	2.81	1.62 (1.50–1.74)^b^	4390 (12.9)	1.00	1114 (6.5)	0.47	0.62 (0.57–0.68)^b^
Preterm birth	2490 (23.3)	3.17	3.16 (2.98–3.36)^c^	2991 (8.8)	1.00	718 (4.2)	0.46	0.44 (0.40–0.48)^c^
LGA	523 (4.9)	0.63	0.60 (0.54–0.66)^c^	2598 (7.6)	1.00	2464 (14.4)	2.05	2.09 (1.97–2.21)^c^
Macrosomia	15 (0.1)	0.34	0.45 (0.26–0.76)^b^	139 (0.4)	1.00	235 (1.4)	3.41	2.72 (2.20–3.37)^b^
GDM	755 (7.1)	2.17	2.08 (1.89–2.29)^f^	1159 (3.4)	1.00	408 (2.4)	0.7	0.72 (0.64–0.81)^f^
Pregnancy-Induced Hypertension	476 (4.5)	1.20	0.70 (0.62–0.78)^g^	1283 (3.8)	1.00	914 (5.3)	1.45	1.68 (1.54–1.84)^g^
PPH with VD	1837 (17.2)	0.79	0.97 (0.92–1.03)^d^	7104 (20.8)	1.00	4215 (24.7)	1.25	1.11 (1.07–1.16)^d^
PPH with CS	870 (8.1)	1.16	0.70 (0.64–0.77)^h^	2431 (7.1)	1.00	1304 (7.6)	1.08	1.36 (1.26–1.46)^h^

BMI, Body Mass Index; SGA, small for gestational age; LGA, large for gestational age; GDM, Gestational Diabetes Mellitus; PPH with VD, postpartum hemorrhage with vaginal delivery; PPH with CS, postpartum hemorrhage with cesarean section; GWG, gestational weight gain;

^a^Adjusting for pre-pregnancy BMI, age, smoking, delivery method.

^b^Adjusting for pre-pregnancy BMI, age, nulliparity, gestational week, smoking, delivery method.

^c^Adjusting for pre-pregnancy BMI, age, nulliparity, smoking, delivery method.

^d^Adjusting for pre-pregnancy BMI, nulliparity, gestational week, smoking.

^e^Adjusting for pre-pregnancy BMI, age, gestational week.

^f^Adjusting for pre-pregnancy BMI, age, smoking.

^g^Adjusting for pre-pregnancy BMI, age, nulliparity, gestational week, delivery method.

^h^Adjusting for pre-pregnancy BMI, age, nulliparity, gestational wek.

Table 3 shows the risks of various pregnancy outcomes associated with weight gain below or above 5 kg in overweight and obese women. The risks of SGA, LBW, and preterm birth were significantly increased with ≤ 5 kg weight gain, whereas the risks of LGA, macrosomia (all adjusted *p* < 0.0001, except for overweight women), pregnancy-induced hypertension, and PPH-CS (all adjusted *p* < 0.0001, except for obese II women) increased significantly with GWG > 5 kg.

**Table 3:** Risks of pregnancy outcomes in overweight and obese women according to weight gain below or above 5 kg.

Overweight women (n = 9,985): pre-pregnancy BMI 23–24.9 kg/m^2^	Weight gain during pregnancy recommended by Japanese Ministry of Health, Labour, and Welfare					
	≤5 kg (n = 1,506)			5 < kg (n = 8,479)		
	n (%)	Crude OR	Adjusted OR (95% CI)	n (%)	Crude OR	Adjusted OR (95% CI)
**Risks associated with insufficient gestational weight gain**						
SGA	195 (13.0)	2.3	2.05 (1.70–2.46)^a^	511 (6.0)	1	—
Low birth weight	386 (25.6)	2.95	1.97 (1.62–2.40)^b^	886 (10.5)	1	—
Preterm birth	326 (21.7)	2.77	2.84 (2.45–3.29)^c^	768 (9.1)	1	—
**Risks associated with excess gestational weight gain**						
LGA	114 (7.6)	1	—	1168 (13.8)	2.0	1.87 (1.52–2.29)^d^
Macrosomia	6 (0.4)	1	—	96 (1.1)	2.86	2.18 (0.95–5.00)^a^
GDM	183 (12.2)	1	—	511 (6.0)	0.46	0.49 (0.41–0.59)^e^
Pregnancy-Induced Hypertension	98 (6.5)	1	—	576 (6.8)	1.05	1.50 (1.17–1.91)^d^
PPH with VD	277 (18.4)	1	—	1863 (22.0)	1.25	1.01 (0.87–1.17)^f^
PPH with CS	158 (10.5)	1	—	814 (9.6)	0.91	1.37 (1.13–1.67)^g^
**Obese I (n = 7,873): pre-pregnancy BMI 25–29.9 kg/m**^**2**^	**Weight gain during pregnancy recommended by Japanese Ministry of Health, Labour, and Welfare**					
	**≤5 kg (n = 2,236)**			**5 < kg (n = 5,637)**		
	**n (%)**	**Crude OR**	**Adjusted OR (95%CI)**	**n (%)**	**Crude OR**	**Adjusted OR (95%CI)**
**Risks associated with insufficient gestational weight gain**						
SGA	222 (9.9)	1.77	1.55 (1.29–1.87)^h^	331 (5.9)	1	—
Low birth weight	513 (23.0)	2.61	1.74 (1.45–2.09)^b^	578 (10.3)	1	—
Preterm birth	416 (18.6)	2.35	2.45 (2.12–2.83)^i^	499 (8.9)	1	—
**Risks associated with excess gestational weight gain**						
LGA	225 (10.1)	1	—	1070 (19.0)	2.10	2.04 (1.75–2.39)^j^
Macrosomia	20 (0.9)	1	—	124 (2.2)	2.49	2.19 (1.35–3.55)^f^
GDM	425 (19.0)	1	—	611 (10.8)	0.52	0.57 (0.50–0.65)^k^
Pregnancy-Induced Hypertension	221 (9.9)	1	—	595 (10.6)	1.08	1.49 (1.25–1.78)^d^
PPH with VD	410 (18.3)	1	—	1247 (22.1)	1.27	1.06 (0.93–1.21)^f^
PPH with CS	262 (11.7)	1	—	648 (11.5)	0.98	1.28 (1.09–1.51)^b^
**Obese II (n = 3,136): pre-pregnancy BMI 30 ≤ kg/m**^**2**^	**Weight gain during pregnancy recommended by Japanese Ministry of Health, Labour, and Welfare**					
	**≤5 kg (n = 1,581)**			**>5 kg (n = 1,555)**		
	**n (%)**	**Crude OR**	**Adjusted OR (95% CI)**	**n (%)**	**Crude OR**	**Adjusted OR (95% CI)**
**Risks associated with insufficient gestational weight gain**						
SGA	145 (9.2)	1.84	1.66 (1.25–2.22)^l^	81 (5.2)	1	—
Low birth weight	277 (17.5)	2.04	1.53 (1.15–2.05)^f^	147 (9.5)	1	—
Preterm birth	254 (16.1)	1.95	2.06 (1.64–2.58)^c^	139 (8.9)	1	—
**Risks associated with excess gestational weight gain**						
LGA	268 (17.0)	1	—	420 (27.0)	1.81	1.84 (1.54–2.20)^m^
Macrosomia	30 (1.9)	1	—	72 (4.6)	2.51	2.40 (1.54–3.75)^a^
GDM	470 (29.7)	1	—	331 (21.3)	0.64	0.69 (0.58–0.81)^e^
Pregnancy-Induced Hypertension	245 (15.5)	1	—	256 (16.5)	1.08	1.29 (1.05–1.58)^d^
PPH with VD	344 (21.8)	1	—	350 (22.5)	1.04	0.90 (0.76–1.08)^b^
PPH with CS	223 (14.1)	1	—	214 (13.8)	0.97	1.14 (0.93–1.41)^g^

*Japanese Ministry of Labour, Health, and Welfare recommends up to 5 kg but depending on individual cases.

BMI, Body Mass Index; SGA, small for gestational age; LGA, large for gestational age; GDM, Gestational Diabetes Mellitus; PPH with VD, postpartum hemorrhage with vaginal delivery; PPH with CS, postpartum hemorrhage with cesarean section; GWG, gestational weight gain;

^a^Adjusting for pre-pregnancy BMI, nulliparity, gestational week, delivery method.

^b^Adjusting for pre-pregnancy BMI, age, nulliparity, gestational week.

^c^Adjusting for pre-pregnancy BMI, nulliparity, delivery method.

^d^Adjusting for pre-pregnancy BMI, age, nulliparity, gestational week, delivery method.

^e^Adjusting for pre-pregnancy BMI, age, smoking.

^f^Adjusting for pre-pregnancy BMI, nulliparity, gestational week.

^g^Adjusting for pre-pregnancy BMI, age, gestational week.

^h^Adjusting for pre-pregnancy BMI, nulliparity, gestational week, smoking, delivery method.

^i^Adjusting for pre-pregnancy BMI, smoking, delivery method.

^j^Adjusting for pre-pregnancy BMI, gestational week, smoking, delivery method.

^k^Adjusting for pre-pregnancy BMI, age, nulliparity, smoking.

^l^Adjusting for pre-pregnancy BMI, gestational week.

^m^Adjusting for pre-pregnancy BMI, gestational week, delivery method.

The risk of GDM decreased significantly with weight gain > 12 kg in normal-weight women [OR 0.72, 95% confidence interval (CI): 0.64–0.81], and with weight gain > 5 kg in overweight women (OR 0.49, 95% CI: 0.41–0.59), obese I women (OR 0.57, 95% CI: 0.50–0.65) and obese II women (OR 0.69, 95% CI: 0.58–0.81).

Table 4 shows the GWG cutoff points based on the area under the curve (AUC) according to various pregnancy outcomes. After adjusting for covariates selected in stepwise multivariable logistic regression analyses, pregnancy outcomes with the lower boundary of a 95% CI of AUC > 0.6 included SGA, preterm birth, LGA, and macrosomia in underweight women; preterm birth, LGA, and macrosomia in normal-weight women; preterm birth, LGA, and macrosomia in overweight women; preterm birth, LGA, and macrosomia in obese I women; and macrosomia in obese II women. Based on the largest Youden’s index calculated by bootstrapped sensitivity and specificity, we estimated the adjusted GWG cutoff points to be 10 kg for SGA and preterm birth, 12 kg for LGA, and 13.8 kg for macrosomia, (i.e., range 10–13.8 kg) in underweight women; to be 10 kg for preterm birth, 11.7 kg for LGA, and 13.7 kg for macrosomia, (i.e., range 10–13.7 kg in normal-weight women; to be 8.5 kg for preterm birth, 11.3 kg for LGA, and 11.4 kg for macrosomia, (i.e., range 8.5–11.4 kg) in overweight women; to be 5 kg for preterm birth, 8 kg for LGA, and 13.3 kg for macrosomia, (i.e., range 5–13.3 kg) in obese I women; and to be 4.7 kg for macrosomia in obese II women.

**Table 4:** Gestational weight gain cutoff points based on maximized area under curve according to pregnancy outcomes.

Asian specific BMI categories	Outcome	Crude	Adjusted										
		GWG cutoff	GWG cutoff	p-value of continuous GWG	AUC	95% CI		Sensitivity	95% CI		Specificity	95% CI	
Underweight	**SGA**^**a**^	9.4	**10**	<0.01	**0.63**	**0.61**	**0.64**	**0.58**	**0.56**	**0.60**	**0.62**	**0.59**	**0.63**
BMI < 18.5 kg/m^2^	LBW^b^	9.1	11.6	<0.01	0.61	0.59	0.62	0.56	0.53	0.58	0.60	0.57	0.63
(n = 18,382)	**PTB**^**c**^	9.4	**10**	<0.01	**0.72**	**0.71**	**0.73**	**0.66**	**0.64**	**0.68**	**0.67**	**0.64**	**0.68**
	**LGA**^**d**^	11.1	**12**	<0.01	**0.69**	**0.67**	**0.71**	**0.66**	**0.62**	**0.68**	**0.64**	**0.59**	**0.66**
	**Macrosomia**^**e**^	13.8	**13.8**	<0.01	**0.78**	**0.70**	**0.85**	**0.67**	**0.53**	**0.80**	**0.81**	**0.49**	**0.88**
	GDM^f^	8.7	8	<0.01	0.61	0.58	0.63	0.42	0.38	0.46	0.75	0.70	0.78
	PIH^g^	12	11.6	<0.01	0.60	0.58	0.63	0.40	0.36	0.44	0.77	0.71	0.79
	PPHVD^h^	10.4	10.3	<0.01	0.53	0.52	0.54	0.54	0.52	0.56	0.50	0.48	0.52
	PPHCS^i^	15.5	10.5	<0.01	0.60	0.58	0.62	0.65	0.61	0.68	0.50	0.46	0.52
Normal weight	SGA^j^	9.8	9.2	<0.01	0.60	0.59	0.60	0.54	0.52	0.55	0.61	0.59	0.62
18.5–23 kg/m^2^	LBW^b^	9.1	11.5	<0.01	0.57	0.56	0.58	0.55	0.53	0.56	0.56	0.55	0.57
(n = 61,960)	**PTB**^**c**^	8.3	**10**	<0.01	**0.70**	**0.69**	**0.71**	**0.59**	**0.57**	**0.60**	**0.71**	**0.70**	**0.73**
	**LGA**^**c**^	10.9	**11.7**	<0.01	**0.64**	**0.63**	**0.64**	**0.51**	**0.50**	**0.52**	**0.69**	**0.67**	**0.70**
	**Macrosomia**^b^	11.3	**13.7**	<0.01	**0.70**	**0.67**	**0.72**	**0.61**	**0.56**	**0.66**	**0.69**	**0.63**	**0.73**
	GDM^f^	7.8	8.5	<0.01	0.61	0.59	0.62	0.44	0.42	0.46	0.73	0.71	0.74
	PIH^g^	12.6	13.3	<0.01	0.60	0.59	0.61	0.44	0.42	0.45	0.72	0.69	0.74
	PPHVD^h^	10.4	10.8	<0.01	0.52	0.51	0.52	0.40	0.39	0.41	0.63	0.61	0.63
	PPHCS^k^	6.8	10.3	<0.01	0.57	0.56	0.58	0.55	0.53	0.56	0.56	0.54	0.57
Overweight	SGA^l^	7.3	9.7	<0.01	0.60	0.58	0.62	0.54	0.50	0.58	0.61	0.57	0.64
23–24.9 kg/m^2^	LBW^k^	6.1	7.3	<0.01	0.59	0.56	0.61	0.59	0.55	0.63	0.53	0.48	0.57
(n = 9,985)	**PTB**^**m**^	6.2	**8.5**	<0.01	**0.65**	**0.64**	**0.67**	**0.58**	**0.54**	**0.61**	**0.66**	**0.62**	**0.69**
	**LGA**^**n**^	8.9	**11.3**	<0.01	**0.62**	**0.61**	**0.64**	**0.55**	**0.52**	**0.58**	**0.63**	**0.59**	**0.65**
	**Macrosomia**^**g**^	10.3	**11.4**	<0.01	**0.66**	**0.61**	**0.71**	**0.72**	**0.63**	**0.79**	**0.54**	**0.42**	**0.60**
	GDM^f^	7.1	8.1	<0.01	0.58	0.56	0.61	0.55	0.51	0.59	0.60	0.55	0.64
	PIH^g^	10	11	<0.01	0.58	0.55	0.60	0.50	0.46	0.54	0.62	0.58	0.65
	PPHVD°	6.4	14.5	<0.01	0.51	0.50	0.53	0.16	0.14	0.18	0.87	0.84	0.88
	PPHCS^i^	6	9.5	<0.01	0.55	0.52	0.56	0.57	0.52	0.60	0.52	0.48	0.54
Obese I	SGA^j^	3.2	6	<0.01	0.57	0.54	0.59	0.32	0.27	0.36	0.80	0.73	0.82
25–29.9 kg/m^2^	LBW^e^	3.1	7.7	<0.01	0.60	0.57	0.63	0.51	0.45	0.55	0.65	0.59	0.68
(n = 7,873)	**PTB**^**p**^	5.3	**5**	<0.01	**0.63**	**0.61**	**0.65**	**0.51**	**0.48**	**0.55**	**0.71**	**0.66**	**0.73**
	**LGA**^**q**^	8.5	**8**	<0.01	**0.62**	**0.60**	**0.63**	**0.54**	**0.50**	**0.56**	**0.64**	**0.60**	**0.66**
	**Macrosomia**^**k**^	6.8	**13.3**	<0.01	**0.65**	**0.60**	**0.69**	**0.39**	**0.30**	**0.47**	**0.84**	**0.75**	**0.88**
	GDM^n^	2.6	5.6	<0.01	0.58	0.56	0.60	0.44	0.40	0.47	0.69	0.66	0.73
	PIH^g^	7	12	<0.01	0.59	0.57	0.61	0.37	0.33	0.41	0.77	0.73	0.79
	PPHVD°	9.6	9.1	<0.01	0.51	0.50	0.53	0.46	0.43	0.48	0.57	0.54	0.60
	PPHCS^k^	8.5	7.2	<0.01	0.54	0.52	0.56	0.58	0.54	0.62	0.49	0.45	0.52
Obese II	SGA^r^	3.2	4.1	<0.01	0.58	0.54	0.62	0.53	0.45	0.59	0.61	0.52	0.66
30 ≤ kg/m^2^	LBW°	3.1	3	<0.01	0.57	0.52	0.61	0.46	0.38	0.53	0.64	0.54	0.71
(n = 3,136)	PTB^s^	5.1	4.5	<0.01	0.61	0.58	0.64	0.56	0.49	0.60	0.63	0.55	0.66
	LGA^r^	8.5	3	<0.01	0.61	0.59	0.64	0.75	0.71	0.78	0.42	0.36	0.45
	**Macrosomia**^**l**^	6.8	**4.7**	<0.01	**0.67**	**0.62**	**0.72**	**0.81**	**0.70**	**0.87**	**0.44**	**0.33**	**0.48**
	GDM^f^	2.6	6.4	<0.01	0.56	0.54	0.59	0.51	0.46	0.55	0.60	0.55	0.63
	PIH^g^	7	11.2	<0.01	0.55	0.52	0.58	0.31	0.25	0.35	0.80	0.74	0.82
	PPHVD^e^	9.6	6	0.10	0.52	0.49	0.55	0.54	0.49	0.58	0.51	0.45	0.54
	PPHCS^e^	8.5	5	0.00	0.54	0.51	0.57	0.63	0.57	0.67	0.45	0.37	0.49

AUC, Area under curve, BMI, Body Mass Index; SGA, small for gestational age; LGA, large for gestational age; LBW, low birth weight; PTB, preterm birth; GDM, Gestational Diabetes Mellitus; PIH, pregnancy-induced hypertension; PPH with VD, postpartum hemorrhage with vaginal delivery; PPH with CS, postpartum hemorrhage with cesarean section; GWG, gestational weight gain, 95%CI, 95%confidence Interval.

^a^Adjusting for pre-pregnancy BMI, age, gestational week, smoking, delivery method.

^b^Adjusting for pre-pregnancy BMI, age, nulliparity, gestational week, smoking, delivery method.

^c^Adjusting for pre-pregnancy BMI, age, nulliparity, smoking, delivery method.

^d^Adjusting for pre-pregnancy BMI, age, nulliparity, gestational week, smoking.

^e^Adjusting for pre-pregnancy BMI, age, gestational week, nulliparity.

^f^Adjusting for pre-pregnancy BMI, age, smoking.

^g^Adjusting for pre-pregnancy BMI, age, nulliparity, gestational week, delivery method.

^h^Adjusting for pre-pregnancy BMI, smoking, nulliparity, gestational week.

^i^Adjusting for pre-pregnancy BMI, age, gestational week.

^j^Adjusting for pre-pregnancy BMI, nulliparity, gestational week, smoking, delivery method.

^k^Adjusting for pre-pregnancy BMI, age, nulliparity, gestational week.

^l^Adjusting for pre-pregnancy BMI, nulliparity, gestational week, delivery method.

^m^Adjusting for pre-pregnancy BMI, nulliparity, smoking, delivery method.

^n^Adjusting for pre-pregnancy BMI, age, nulliparity, smoking.

^o^Adjusting for pre-pregnancy BMI, nulliparity, gestational week.

^p^Adjusting for pre-pregnancy BMI, smoking, delivery method.

^q^Adjusting for pre-pregnancy BMI, age, gestational week, smoking.

^r^Adjusting for pre-pregnancy BMI, gestational week.

^s^Adjusting for pre-pregnancy BMI, nulliparity, delivery method.

Table 5 shows the results of sensitivity analyses when women with preterm birth were excluded. The cutoff points for GWG with a lower boundary of 95% CI of the adjusted AUC > 0.6 corresponded to 11.2 kg for SGA, 10 kg for LBW, 12.6 kg for LGA, and 12 kg for macrosomia, (i.e., range 10–12.6 kg) in underweight women; to 10.6 kg for SGA, 10.4 kg for LBW, 10 kg for LGA, and 12.7 kg for macrosomia, (i.e., range 10–12.7 kg) in normal-weight women; to 11.3 kg for LGA, 10 kg for macrosomia, (i.e., range 11.3–11.4 kg) in overweight women, 11.5 kg for LGA, and 14.1 kg for macrosomia, (i.e., range 11.5–14.1 kg) in obese I women; and to 5.8 kg for macrosomia in obese II women.

**Table 5:** Gestational weight gain cutoff points according to SGA, LBW, LGA, and macrosomia excluding women with preterm birth.

Asian specific BMI categories	Outcome	Crude	Adjusted										
		GWG cutoff	GWG cutoff	p-value of continuous GWG	AUC	95% CI		Sensitivity	95% CI		Specificity	95% CI	
Underweight	**SGA**^**a**^	9.4	**11.2**	<0.01	**0.62**	**0.62**	**0.63**	**0.55**	**0.53**	**0.56**	**0.64**	**0.62**	**0.65**
BMI < 18.5 kg/m^2^	**LBW**^**a**^	9.5	**10**	<0.01	**0.62**	**0.61**	**0.63**	**0.54**	**0.52**	**0.55**	**0.65**	**0.63**	**0.66**
(n = 16,177)	**LGA**^**b**^	11.5	**12.6**	<0.01	**0.64**	**0.63**	**0.65**	**0.54**	**0.53**	**0.55**	**0.67**	**0.66**	**0.68**
	**Macrosomia**^**c**^	13	**12**	<0.01	**0.69**	**0.67**	**0.71**	**0.56**	**0.52**	**0.59**	**0.73**	**0.69**	**0.76**
Normal weight	**SGA**^**d**^	9.7	**10.6**	<0.01	**0.62**	**0.61**	**0.62**	**0.53**	**0.51**	**0.55**	**0.64**	**0.62**	**0.66**
18.5–23 kg/m^2^	**LBW**^**a**^	9.5	**10.4**	<0.01	**0.61**	**0.60**	**0.62**	**0.63**	**0.61**	**0.65**	**0.54**	**0.51**	**0.55**
(n = 55,761)	**LGA**^**a**^	10.9	**10**	<0.01	**0.61**	**0.60**	**0.62**	**0.63**	**0.61**	**0.65**	**0.54**	**0.51**	**0.55**
	**Macrosomia**^**a**^	11.3	**12.7**	<0.01	**0.70**	**0.67**	**0.72**	**0.61**	**0.56**	**0.66**	**0.69**	**0.64**	**0.72**
Overweight	SGA^e^	9.7	10	<0.01	0.62	0.59	0.64	0.66	0.61	0.70	0.52	0.47	0.55
23–24.9 kg/m^2^	LBW^f^	9.9	7.5	<0.01	0.61	0.58	0.63	0.40	0.35	0.45	0.74	0.68	0.78
(n = 8,891)	**LGA**^**g**^	10.6	**11.3**	<0.01	**0.62**	**0.61**	**0.64**	**0.55**	**0.52**	**0.58**	**0.63**	**0.59**	**0.66**
	**Macrosomia**^**h**^	11.4	**11.4**	<0.01	**0.66**	**0.61**	**0.72**	**0.73**	**0.61**	**0.79**	**0.54**	**0.42**	**0.59**
Obese I	SGA^i^	3.5	7	<0.01	0.59	0.56	0.62	0.53	0.47	0.58	0.61	0.53	0.65
25–29.9 kg/m^2^	LBW^c^	5.4	7.5	<0.01	0.60	0.57	0.63	0.53	0.47	0.57	0.63	0.57	0.67
(n = 6,958)	**LGA**^**g**^	8.9	**11.5**	<0.01	**0.62**	**0.60**	**0.64**	**0.42**	**0.39**	**0.45**	**0.75**	**0.71**	**0.77**
	**Macrosomia**^**c**^	10.3	**14.1**	<0.01	**0.65**	**0.61**	**0.70**	**0.40**	**0.30**	**0.47**	**0.85**	**0.75**	**0.88**
Obese II	SGA^j^	3.1	3.4	<0.01	0.59	0.54	0.64	0.45	0.34	0.53	0.72	0.61	0.76
30 ≤ kg/m^2^	LBW^f^	1.4	3.5	<0.01	0.61	0.56	0.65	0.54	0.45	0.62	0.66	0.52	0.72
(n = 2,743)	LGA^k^	8.5	5	<0.01	0.61	0.59	0.64	0.74	0.69	0.77	0.43	0.39	0.47
	**Macrosomia**^**l**^	6.8	**5.8**	<0.01	**0.66**	**0.60**	**0.71**	**0.81**	**0.68**	**0.87**	**0.43**	**0.33**	**0.48**

AUC, Area under curve, BMI, Body Mass Index; SGA, small for gestational age; LGA, large for gestational age; LBW, low birth weight; GWG, gestational weight gain, 95%CI, 95%Confidence Interval.

^a^Adjusting for pre-pregnancy BMI, age, nulliparity, gestational week, smoking, delivery method.

^b^Adjusting for pre-pregnancy BMI, age, smoking, nulliparity.

^c^Adjusting for pre-pregnancy BMI, age, nulliparity, gestational week.

^d^Adjusting for pre-pregnancy BMI, age, gestational week, smoking, delivery method.

^e^Adjusting for pre-pregnancy BMI, nulliparity.

^f^Adjusting for pre-pregnancy BMI, nulliparity, gestational week.

^g^Adjusting for pre-pregnancy BMI, age, smoking, delivery method.

^h^Adjusting for pre-pregnancy BMI, age, nulliparity, gestational week, delivery method.

^i^Adjusting for pre-pregnancy BMI, nulliparity, smoking.

^j^Adjusting for pre-pregnancy BMI.

^k^Adjusting for pre-pregnancy BMI, age.

^l^Adjusting for pre-pregnancy BMI, nulliparity, gestational week, delivery method.

## Discussion

Utilizing the JSOG Successive Pregnancy Birth Registry, this study showed that the current Japanese GWG recommendations can significantly identify the risks of multiple pregnancy outcomes. Among these outcomes, the risks of SGA, LBW, and preterm birth significantly increased with insufficient weight gain, whereas the risks of LGA and macrosomia significantly increased with excessive weight gain regardless of pre-pregnancy BMI, except for macrosomia in women classified as overweight. The GWG cutoff points estimated by the adjusted AUC > 0.6 corresponded to range 10–13.8 kg in underweight women; to range 10–13.7 kg in normal-weight women; to range 8.5–11.4 kg in overweight women; to range 5–13.3 kg in obese I women; and to be 4.7 kg for macrosomia in obese II women. When women with preterm birth were excluded, these cutoff points for GWG corresponded to range 10–12.6 kg in underweight women; to range 10–12.7 kg in normal-weight women; to range 11.3–11.4 kg in overweight women, range 11.5–14.1 kg in obese I women; and to 5.8 kg for macrosomia in obese II women. The optimal GWG ranges proposed by the present study are slightly higher than those recommended by the current Japanese guidelines: 9–12 kg for underweight and 7–12 kg for normal-weight women, and ≤ 5 kg (depending on individual cases) for overweight and obese women.

**Figure 1:**
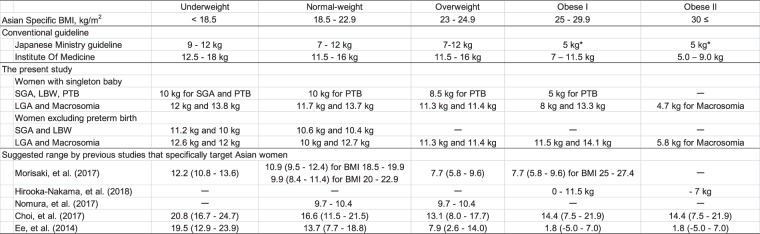
Weight gain during pregnancy recommended by the Institute of Medicine, the Japanese Ministry of Health, Labour, and Welfare, present and previous studies in Asian countries. *Approximately up to 5 kg but depending on individual cases BMI, Body Mass Index; SGA, small for gestational age; LGA, large for gestational age; LBW, low birth weight; PTB, preterm birth.

Several previous studies investigated optimal GWG for Asian women for whom there is no universal standard. The ranges proposed to date have been inconsistent (Fig. 1), with some proposing GWG below^21,24–26^ the IOM recommendations and others proposing GWG above the IOM range^25–27^. These studies used various pregnancy outcomes and evaluation methods with various sample sizes of target populations differing in various respects (e.g., ethnicity, overweight/obese women, limited to full-term pregnancy). For example, Ee *et al*.^26^ investigated 1,529 women (63%, Chinese) and estimated optimal GWG using composite perinatal outcome based on a combination of delivery method and fetal size for gestational age. Choi *et al*.^27^ investigated 3,285 Korean women with full-term singleton births and estimated optimal GWG as not exceeding a 5% increase from the lowest total predicted risks of gestational hypertensive disorders, emergency cesarean section, and fetal size for gestational age. Among these previous studies, Morisaki *et al*.^24^ investigated the largest sample size of 104,070 women who were registered in the Japanese perinatal database in 2005–2011, and developed a predictive model based on another sample of 1,283 mothers at a single hospital. The authors calculated expected the GWG over 40 weeks corresponding to a 1% increase in the risk of preterm delivery, SGA, complicated delivery, and preeclampsia. One study conducted by Hirooka-Nakama *et al*.^25^ used the same dataset as used in this study (similar in size to that used by Morisaki *et al*.^24^) but focused on overweight/obese women only and determined an optimal GWG of 0 kg based on the lowest risk probability of the sum of various pregnancy outcomes and 11. 5 kg, at which point the risk increased sharply. Although it may not be possible to take into account the speed of weight gain per trimester as ROC analysis is based on cross-sectional evaluation, calculating the adjusted cutoff points of individual multi-pregnancy outcomes is simple, straightforward, and easy to understand rather than composite outcomes or using complicated statistical models with various assumptions. We also adopted the cutoff points of GWG at the lower boundary of a 95% CI of AUC > 0.6, and the results indicate that GWG has good predictive capability of a specific pregnancy outcome to determine accurate optimal GWG. Furthermore, we performed sensitivity analyses by limiting the subjects to those with full-term births because the database included large and tertiary hospitals and the subjects registered in the database might have had high risk profiles. If such unhealthy conditions were related to the birth of small babies, the true association might have been overestimated. The results were, however, similar between the models with inclusion or exclusion of preterm birth. Therefore, the results imply that optimal GWG is slightly higher than the current Japanese guidelines, regardless of the inclusion of preterm birth.

In this study, the AUCs had good predictive capability for macrosomia and LGA regardless of pre-pregnancy BMI category. Our ROC analyses showed that the optimal GWG range for large infants (i.e., LGA and macrosomia) exceeded the currently recommended threshold according to the Japanese guidelines (i.e., 5 kg for overweight and obese women). In this regard, the upper boundary of current GWG recommendations may still allow some room for these overweight/obese women to gain weight. In determining the upper boundary of GWG, the risk of hemorrhage at delivery is a major concern, as hemorrhagic obstetric complications are the leading cause of maternal deaths in Japan^28^. However, in the present study, PPH-VD and PPH-CS had low adjusted AUCs, sensitivity, and specificity, implying that weight gain may have little effect on hemorrhage. Our results are consistent with a previous study^29^ indicating that overweight/obese women are more likely to have high incidences of LGA, GDM, and PIH, but these pregnancy outcomes/complications are independent of weight gain, and are therefore difficult to manage by weight gain control. In Japan, obese women are more likely to receive strict instruction to limit weight gain to prevent dystocia^30^, but they might feel more at ease if the upper boundary of the current Japanese guidelines is increased for overweight and obese women (i.e., ~30 kg/m^2^ as implied by the present study in Fig. 1).

We expected that excessive GWG would be associated with an increased risk of GDM, but found the opposite: underweight and normal-weight women with GWG below the recommendations were more likely to have GDM, whereas normal-weight, overweight, and obese women with GWG above the recommendations were less likely to have GDM. These findings are consistent with a previous study^2^ using the same dataset, indicating that there were no errors in our calculations. One explanation may be that GDM is usually diagnosed in the second trimester (i.e., around gestational week 24–28), and it is therefore possible that women with a diagnosis of GDM are monitored more closely and encouraged to improve their behaviors (i.e., diet, physical activity) resulting in limited total GWG. Similarly, two Asian studies of 48,867 Chinese women^31^ and 7,843 Korean women^17^ provide further examples of a counterintuitive relationship between GDM and GWG during pregnancy. The subjects in these studies were recruited at large hospitals, where individual mothers might have had more health concerns, and therefore overweight and obese women in particular were more likely to adhere to weight-gain recommendations. Furthermore, one study compared 4,930 Asian Indians with 2,868 Caucasians and demonstrated that the prevalence of diabetes in underweight subjects was higher in Asians than in Caucasians. Ethnic differences in diabetes susceptibility (Asians > Caucasians)^32^ may also enhance the attention of individuals to strict blood-sugar control during pregnancy.

This study had some limitations. First, due to the database used in this study, all of the variables had some degree of unrealistic data (e.g., maternal height outside the range of 60–200 cm or pre-pregnancy BMI), implying that data reliability may have been low. Although these corresponded to a total of 1,146 subjects constituting <1% of all data, thus reducing their impact on the results, they were excluded to increase the accuracy of the analyses. Second, data on lifestyle factors, including smoking and drinking habits, as well as social factors, including socioeconomic status, were missing. The birth of small babies has been linked to maternal smoking^33^ and poor nutritional status^34^; therefore, it would have been better to adjust for social and environmental factors. Third, pre-pregnancy BMI and GWG were estimated based on self-reporting of weight before pregnancy, which might have been subject to recall bias. Fourth, in this study, we did not investigate preeclampsia as an outcome; however, this exclusion is recognized by the IOM Working Group as there is only weak evidence that excessive weight gain can cause preeclampsia and the reverse association is also plausible, in that edema due to preeclampsia may cause increased weight gain^35^. Finally, the majority of women registered in the JSOG birth database had delivered at tertiary hospitals, indicating that they were more likely to represent a high-risk group. Although we confirmed that the results did not change if we excluded those with preterm birth, further research is needed to assess whether our findings are generalizable to other Japanese populations.

Despite these limitations, the present study based on adjusted ROC curves indicated that the current Japanese recommendations for GWG may be lower than the optimal GWG ranges and might have a merit if both lower and upper boundaries are increased.

## Methods

### Subjects for analyses in the JSOG database

**Figure 2:**
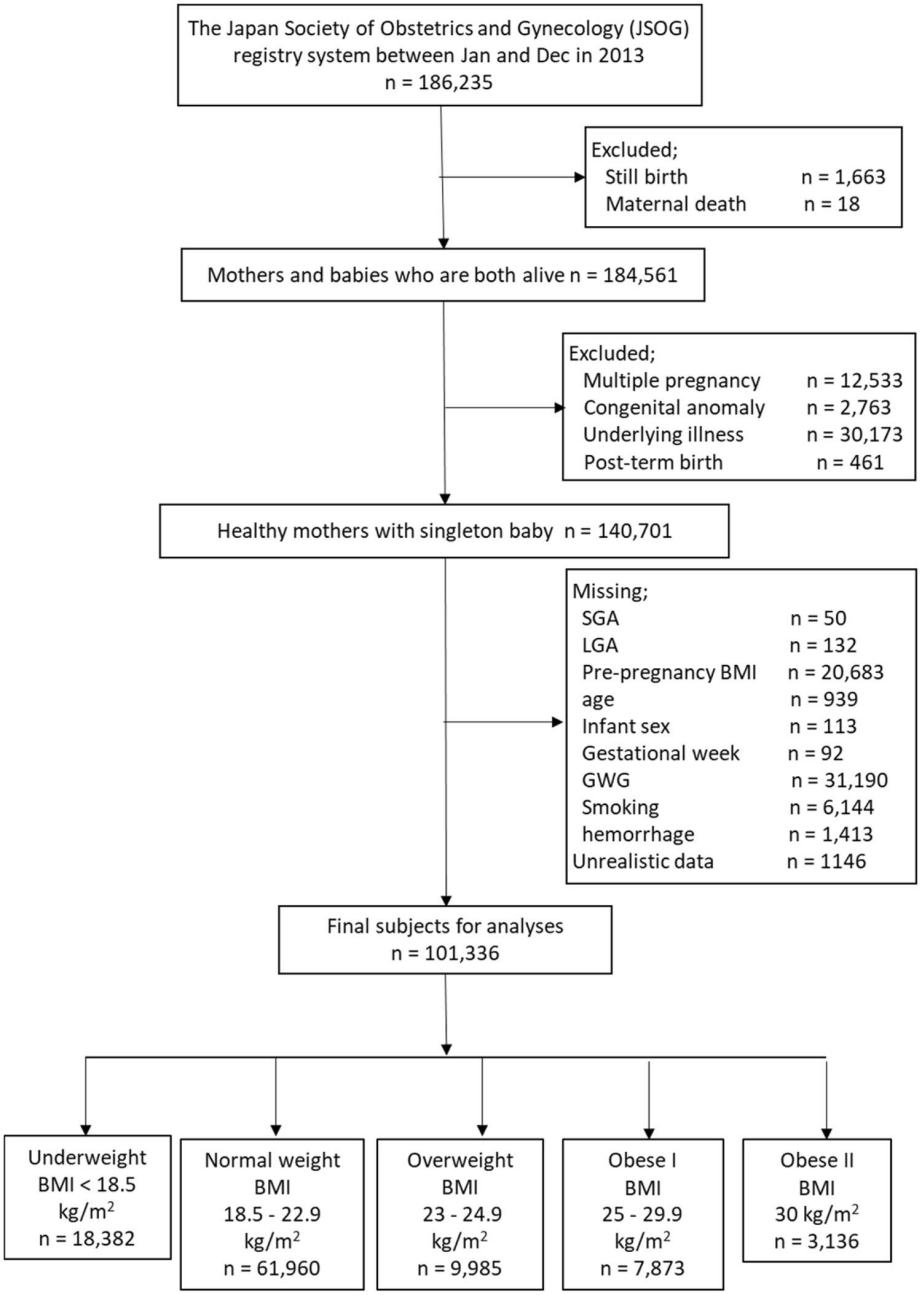
Flow chart of study enrollment.

JSOG developed the Successive Pregnancy Birth Registry System in 2001 to monitor the safety of pregnancy outcomes. This cross-sectional study was conducted after approval from the Ethics Committee of Hamamatsu University School of Medicine. The patient records were anonymized and de-identified prior to analysis and the committee confirmed that the research was performed in accordance with relevant guidelines. Informed consent was obtained in the form of opting out on the web-site at http://www.jsog.or.jp/activity/pdf/Clinical_research_2017–69.pdf. The details have been described in a previous study^2^. Weight gain during pregnancy (i.e., GWG) was calculated by subtracting pre-pregnancy weight from maternal weight at delivery. Briefly, we analyzed 186,235 women who gave birth at gestational week 22 or later between January 1 and December 31, 2013 in approximately 280 secondary and tertiary hospitals. Figure 2 is the flowchart of subjects included in the analyses. After excluding multiple pregnancies (*n* = 12,533), congenital anomalies (*n* = 2,763), underlying illnesses (*n* = 30,173), and post-term births (*n* = 461), the dataset comprised 140,701 women with full-term singleton babies. Among these, missing data on SGA (*n* = 50), LGA (*n* = 132), pre-pregnancy BMI (*n* = 20,683), age (*n* = 939), infant sex (*n* = 113), gestational weeks (*n* = 92), GWG (*n* = 31,190), smoking status (*n* = 6,144), and hemorrhage (*n* = 1,413) were excluded. We further excluded unrealistic data, including cases with recorded birth weight < 500 g (*n* = 211), birth height < 25 cm (*n* = 176), if mother’s maternal weight was outside the range 20–200 kg (*n* = 105), if mother’s maternal height was outside the range 60–200 cm (*n* = 156), if pre-pregnancy BMI was outside the range 6–50 kg/m^2^ (*n* = 254), GWG < −8 kg or > 40 kg (*n* = 767), parity number exceeding 30 (*n* = 2), and infant head circumference exceeding 66.5 cm (*n* = 122). A total of 1,146 subjects showed such unrealistic data. Ultimately, 101,336 subjects were included in our analyses. In 2000, the WHO redefined the BMI classification for the Asian population^22^, proposing the criterion for obesity as BMI > 25 kg/m^2^ and that for overweight status as BMI 23–25 kg/m^2^ for people from the Asia-Oceania region. In this study, based on the Asian-specific pre-pregnancy BMI classification recommended by the WHO, 18,382 women were underweight with BMI < 18.5 kg/m^2^, 61,960 women were normal weight with BMI 18.5–22.9 kg/m^2^, 9,985 women were overweight with BMI 23–24.9 kg/m^2^, 7,873 women were obese with BMI 25–29.9 kg/m^2^, and 3,136 women were obese with BMI ≥ 30 kg/m^2^.

### Pregnancy outcomes

We determined pregnancy outcomes, including SGA (<10th percentile of infant growth curve)^36^, LGA (>90th percentile of infant growth curve)^36^, LBW (<2,500 g), macrosomia (birth weight >4,000 g), preterm birth (<37 gestational weeks), GDM (based on 75-g oral glucose tolerance test and at least one of the following: ≥92 mg/dL fasting glucose, ≥180 mg/dL glucose at 1 h, or ≥153 mg/dL glucose at 2 h), pregnancy-induced hypertension (systolic blood pressure ≥140 and/or diastolic blood pressure ≥90 mmHg after 20 weeks of gestation); PPH-VD (blood loss ≥500 mL), and PPH-CS (blood loss ≥1,000 mL).

### Covariates

The items investigated in this study included maternal age, parity, gestational week at delivery, height, pre-pregnancy body weight, body weight at delivery, delivery mode, sex of infant, and birth weight. Gestational weeks were determined based on the last menstrual period.

### Statistical analyses

All analyses conducted were stratified by Asian-specific BMI classification (i.e., <18.5 kg/m^2^ for underweight, 18.5–22.9 kg/m^2^ for normal weight, 23–24.9 kg/m^2^ for overweight, 25–29.9 kg/m^2^ for obese I, 30 < kg/m^2^ for obese II)^22^. To assess the applicability of the current Japanese recommendations for GWG, we estimated the risks of pregnancy outcomes associated with GWG below and above the range recommended in the Japanese guidelines compared to GWG within the recommended range in underweight (i.e., 9–12 kg) and normal-weight women (i.e., 7–12 kg). For overweight and obese women, the guidelines recommend weight gain of up to 5 kg depending on individual characteristics. Accordingly, we calculated odds ratios for various pregnancy outcomes below and above a weight gain of 5 kg in overweight and obese women. We used a logistic regression model to calculate ORs together with 95% CIs within each BMI category, after adjusting for covariates. The covariates included maternal age, pre-pregnancy BMI, parity, delivery method, smoking, and gestational week, and were selected by each stepwise multivariable model. Among these, as maternal weight is a risk factor for the outcomes of interest, continuous pre-pregnancy BMI as well as GWG classification were included in stepwise models. For the outcome of preterm birth, as it was defined based on gestational week <37 and was strongly correlated with gestational week, gestational week was excluded from the covariates in the selection models. Similarly, as the outcomes PPH-VD and PPH-CS were strongly explained by delivery method, these variables were also excluded from the covariates in the selection models.

To estimate the risk of any adverse pregnancy outcome based on pre-pregnancy BMI levels, crude ROC curves and adjusted ROC curves were drawn adjusting for covariates selected in stepwise multivariable logistic regression models to investigate the risks of various pregnancy outcomes associated with the continuous GWG variable. The covariate-adjusted ROC curves and AUC were calculated based on the method of Pepe *et al*.^37^. The AUC is considered an effective measure of accuracy with a meaningful interpretation, and AUC values of 0.6–0.8 are considered acceptable for prediction of GWG based on Hosmer and Lemeshow^38^. In determining the optimal GWG range, we calculated Youden’s index as sensitivity + specificity - 1 based on the reliable sensitivity and specificity estimated using the bootstrap method^39^. Youden’s index indicates the maximum vertical distance of the ROC curve from a point (x, y) on the diagonal line, and thus maximizes the difference between the true positive fraction and false positive fraction^23^. We calculated the adjusted cutoff points of GWG corresponding to the largest Youden’s index value and determined the optimal GWG for a pregnancy outcome with its lower boundary of the 95% CI of AUC > 0.6. We performed sensitivity analyses both including and excluding women with preterm birth to estimate optimal GWG ranges.

All data were analyzed using SAS version 9.4 for Windows (SAS Institute, Cary, NC) and Stata version 14.2 (Stata Corp., College Station, TX). In all analyses, *P* < 0.05 was taken to indicate statistical significance.
